# Supplementary material for: Complexome profiling of the Chlamydomonas psb28 mutant reveals TEF5 as an early PSII assembly factor
Source: Plant Cell. 2025 Mar 17;37(6):koaf055. doi: 10.1093/plcell/koaf055 (PMC12164590; doi:10.1093/plcell/koaf055)
Supplement: koaf055_Supplementary_Data [file koaf055_supplementary_data.zip › psb28_interactive.zip!/plots/intensity/qualityPlots/peptides/_Cre02.g079400.t1.2.html]

### Cre02.g079400.t1.2

Trivial name:   
SeqCoverage [median]: 29.50  
Mass: 15.82 kDa  
  
 

| Sequence | MissedCleavage | Mass [Da] | wtIntensity | muIntensity | Score |
| --- | --- | --- | --- | --- | --- |
| ENDGKDFMGR | 1 | 1167.50 | 0.00 | 317.81 | 61.16 |
| GMAFVNVDPEKMDAAIR | 1 | 1862.90 | 0.00 | 1251.46 | 57.33 |
| SQAAAPTAPAEAQPSQ | 0 | 1523.72 | 0.00 | 1367.54 | 114.72 |
|  |  |  | 0.0 | 2936.8 |  |
